# Emery-Dreifuss muscular dystrophy and familial partial lipodystrophy, Dunnigan variety due to heterozygous *LMNA* variants

**DOI:** 10.1210/jendso/bvag128

**Published:** 2026-06-05

**Authors:** Anum, Xilong Li, Rebecca J Brown, Abhimanyu Garg

**Affiliations:** The Section of Nutrition and Metabolic Diseases, Division of Endocrinology, Department of Internal Medicine and the Center for Human Nutrition, UT Southwestern Medical Center, Dallas, TX 75390-8537, USA; Peter O’Donnell Jr. School of Public Health, UT Southwestern Medical Center, Dallas, TX 75390-9066, USA; National Institute of Diabetes and Digestive and Kidney Diseases, National Institutes of Health, Bethesda, MD 20892, USA; The Section of Nutrition and Metabolic Diseases, Division of Endocrinology, Department of Internal Medicine and the Center for Human Nutrition, UT Southwestern Medical Center, Dallas, TX 75390-8537, USA

**Keywords:** Emery-Dreifuss muscular dystrophy type 2, lamin A/C, familial partial lipodystrophy, Dunnigan-type, *LMNA*

## Abstract

**Context:**

Specific heterozygous pathogenic variants in *LMNA* have been reported in patients with Dunnigan-type familial partial lipodystrophy (FPLD2), while other variants cause autosomal dominant Emery-Dreifuss muscular dystrophy (AD-EDMD). FPLD2 is characterized by variable loss of subcutaneous fat from the extremities and trunk, and AD-EDMD is characterized by early joint contractures, proximal muscle weakness, and cardiac conduction defects. Previously, only 2 families have been reported with combined features of FPLD2 and AD-EDMD.

**Objective:**

To report the overlapping phenotype of both FPLD2 and AD-EDMD in 5 families with heterozygous *LMNA* variants.

**Methods:**

Clinical, anthropometric, laboratory, and genotyping data of affected subjects from 5 families with female probands presenting with FPLD2 and AD-EDMD phenotypes were collected.

**Results:**

Affected individuals (8 females, ages 18-57 years; 3 males, ages 25-41 years) harbored heterozygous p.T528R, p.R527P, p.R541P, or p.R453W *LMNA* variants. Five females and 2 males had joint contractures and proximal muscle weakness, and 4 females and 1 male had pacemaker-defibrillator implantation for cardiac arrhythmias, confirming AD-EDMD. Subcutaneous fat loss from the extremities confirming FPLD2 was observed in all females but only in 2 males. Four females had diabetes mellitus and hypertriglyceridemia, and 1 male had hypertriglyceridemia. Two females, 50 and 58 years old, died due to aspiration pneumonia and cerebrovascular accident, respectively, and one 40-year-old male died of alcoholic liver disease.

**Conclusion:**

Our report brings attention to the frequent co-occurrence of FPLD2 and AD-EDMD. In the future, all patients with AD-EDMD should be carefully evaluated for clinical signs of lipodystrophy and metabolic complications.

In the 1960s, Emery and Dreifuss [1] reported an X-linked muscular dystrophy associated with contractures and myocardial involvement in a family, which was thereafter called Emery-Dreifuss muscular dystrophy (EDMD) [2, 3]. Almost 2 decades later, several investigators reported autosomal dominant inheritance in a few families with EDMD [4-6]. Thereafter, using linkage analysis approach in a large French pedigree, Bonne et al [7] mapped the locus for autosomal dominant EDMD (AD-EDMD) on chromosome 1q11-q23 and reported disease-causing missense variants in the lamin A/C (*LMNA*) gene in 4 pedigrees. *LMNA* encodes nuclear lamins A and C, which are intermediate filament proteins associated with the nuclear lamina. Specific heterozygous missense variants in *LMNA* cause a variety of other disorders including dilated cardiomyopathy with conduction system defects [8], limb-girdle muscular dystrophy 1B with atrioventricular conduction disturbances [9], Dunnigan-type familial partial lipodystrophy (FPLD2) [10, 11], atypical progeroid syndrome [12], and Hutchinson-Gilford progeria syndrome [13, 14].

AD-EDMD (OMIM #181350) is characterized by early joint contractures, slowly progressive muscle wasting, and weakness with a distinctive humero-peroneal distribution and cardiac conduction defects [15]. FPLD2 (OMIM # 151660) is characterized by loss of subcutaneous fat from the upper and lower extremities with excess fat accumulation in the face, neck, labia majora, and visceral depots [16]. Our group linked the FPLD2 locus to chromosome 1q21-22 [17], and thereafter, Cao and Hegele [10] and others [18, 19] reported missense variants in *LMNA* in patients with FPLD2. Subsequently, many *LMNA* variants have been reported to be associated with typical and atypical varieties of FPLD2 [20, 21]. Affected individuals with FPLD2 show an increased preponderance of insulin resistance, diabetes mellitus, dyslipidemia, and acanthosis nigricans [22]. Some FPLD2 patients have been reported with multisystem dystrophy with cardiomyopathy and muscle weakness [23, 24]. However, only 2 families with heterozygous *LMNA* variants have been reported with overlapping features of AD-EDMD and FPLD2 [25]. We now report 5 families with overlapping presentation of AD-EDMD as well as FPLD2 due to heterozygous *LMNA* variants.

## Material and methods

All patients and their relatives gave written informed consent. The study protocols were approved by the institutional review boards of the University of Texas Southwestern Medical Center (UTSW) and National Institutes of Health (NIH). Clinical, anthropometric, and laboratory data of all patients and their relatives were collected prospectively as well as retrospectively from their medical charts and lipodystrophy questionnaire.

### Questionnaire

We collected demographic data and healthcare history from patient-completed lipodystrophy questionnaires and interviews. The presence of diabetes mellitus, hypertriglyceridemia, steatotic liver disease, polycystic ovarian syndrome, hypertension, coronary heart disease, and stroke were self-reported by the participants and/or documented in laboratory or imaging studies.

### Anthropometric measurements

Height and body weight were measured by standard procedures. Skinfold thickness was measured with a Lange caliper (Cambridge Scientific Industries, Cambridge, MD) at the following 12 sites: chin, truncal (chest, axillary, abdomen, subscapular, and suprailiac), and peripheral (biceps, triceps, forearm, hip, mid-thigh, and calf) sites on the right side of the body. The average of 3 repeat measurements at each site was calculated.

### Dual-energy X-ray absorptiometry

Total and segmental body fat in the trunk and upper and lower extremities were evaluated by whole-body dual-energy X-ray absorptiometry (DXA). For the UTSW cohort, whole-body DXA examinations were acquired on Discovery W (S/N 84310) model machine according to the procedures recommended by the manufacturer (Hologic, Inc., Bedford, MA). DXA scanning at the NIH was performed on Hologic QDR 4500 (Hologic) scanner using Apex 4.0 software. The proportion of fat in specific body regions as well as whole body was calculated as a percentage of body mass [26, 27]. Mean values of the right and left upper limb fat and right and left lower limb fat (% of regional fat) were calculated. We also calculated the ratio of the lower limb to truncal fat. DXA data from sex- and age-matched controls from the National Health and Nutrition Examination Survey (NHANES) from 4 survey cycles, 1999-2000, 2001-2002, 2003-2004, and 2005-2006 were used for comparison. The NHANES percentiles were calculated using SAS 9.4 (SAS Institute, Cary, NC).

### Blood tests for biochemical parameters

Overnight fasting blood samples were collected and analyzed for laboratory variables. Blood samples of the patients from FPL424 and FPL228 pedigree were sent to Quest Diagnostics (Irving, TX) for analysis. Serum glucose, lipids, high density lipoprotein-cholesterol (HDL-C), creatinine, and liver enzymes were measured by spectrometry method (Beckman Coulter AU clinical analyzer), and blood hemoglobin A1c was measured by the enzymatic method (Roche Integra 800 chemistry analyzer). Serum leptin levels were measured by Quest Diagnostics.

Blood hemoglobin A1c, serum glucose, alanine aminotransferase, and aspartate aminotransferase for patients FPL4400.4, FPL465.3, and FPL466.3 were measured using standard techniques of the NIH Clinical Center Department of Laboratory Medicine. Total cholesterol, HDL-C, and triglycerides were measured on the Roche Cobas 6000 Analyzer (Basel, Switzerland). Low density lipoprotein-cholesterol (LDL-C) was calculated using the Sampson-NIH equation [28]. Serum leptin at the NIH was measured using commercial kits (EMD Millipore, Billerica, MA, USA).

### Definitions and diagnosis of metabolic and cardiac disorders

Diagnosis of diabetes mellitus was based on self-reported history, previous medical records, and laboratory findings, according to American Diabetes Association's criteria [29]. Hypertriglyceridemia was defined as fasting serum triglyceride concentration ≥150 mg/dL, or if the participant was on triglyceride-lowering therapy (fibric acid derivatives and/or high-dose omega-3 fatty acid derivatives containing eicosapentaenoic or docosahexaenoic acid), or self-reported. Low concentration of HDL-C was defined as less than 40 mg/dL and 50 mg/dL in males and females, respectively [30]. The reference ranges for serum creatine kinase were 29 to 143 U/L for females and 44 to 196 U/L for males at Quest Diagnostics and 29 to 168 U/L for females and 30 to 200 U/L for males at the NIH Clinical Center Department of Laboratory Medicine. Any serum creatine kinase value above the upper limit of normal was considered elevated. Hepatic steatosis (fatty liver) was defined based on either pathological or radiologic findings. The prevalence of metabolic complications and metabolic variables were compared between the affected males and females, and a *P*-value of <.05 was considered significant. Fisher's exact test was used for comparison of the categorial variables and the Wilcoxon rank-sum test for the continuous variables.

Information about cardiac and skeletal muscle involvement was obtained by interviews and reviewing medical records that included, when available, 12-lead electrocardiography, cardiac catheterization, coronary angiography, and transthoracic echocardiography. Patients were considered to have cardiac involvement if there was evidence of sinoatrial dysfunction, atrial fibrillation, atrioventricular conduction system abnormalities, pacemaker implantation, left ventricular systolic dysfunction (ejection fraction <45% as determined by transthoracic echocardiography, multiple gated acquisition technique, or ventriculography), or symptomatic heart failure in the absence of other known causes.

### Genetic testing

Genomic DNA was isolated from buffy coat using the Easy-DNA kit (Invitrogen, Carlsbad, CA). The samples were genotyped for *LMNA* using Sanger sequencing at UTSW as reported previously [12]. Sequences were compared using Clustal Omega software and by visual inspection. We further confirmed the pathogenic variants by reverse Sanger sequencing. One patient (FPL466.3) underwent commercial testing at Invitae using Illumina technologies and confirmation of the clinically significant variant by Sanger sequencing.

#### Participants

**Figure 1:**
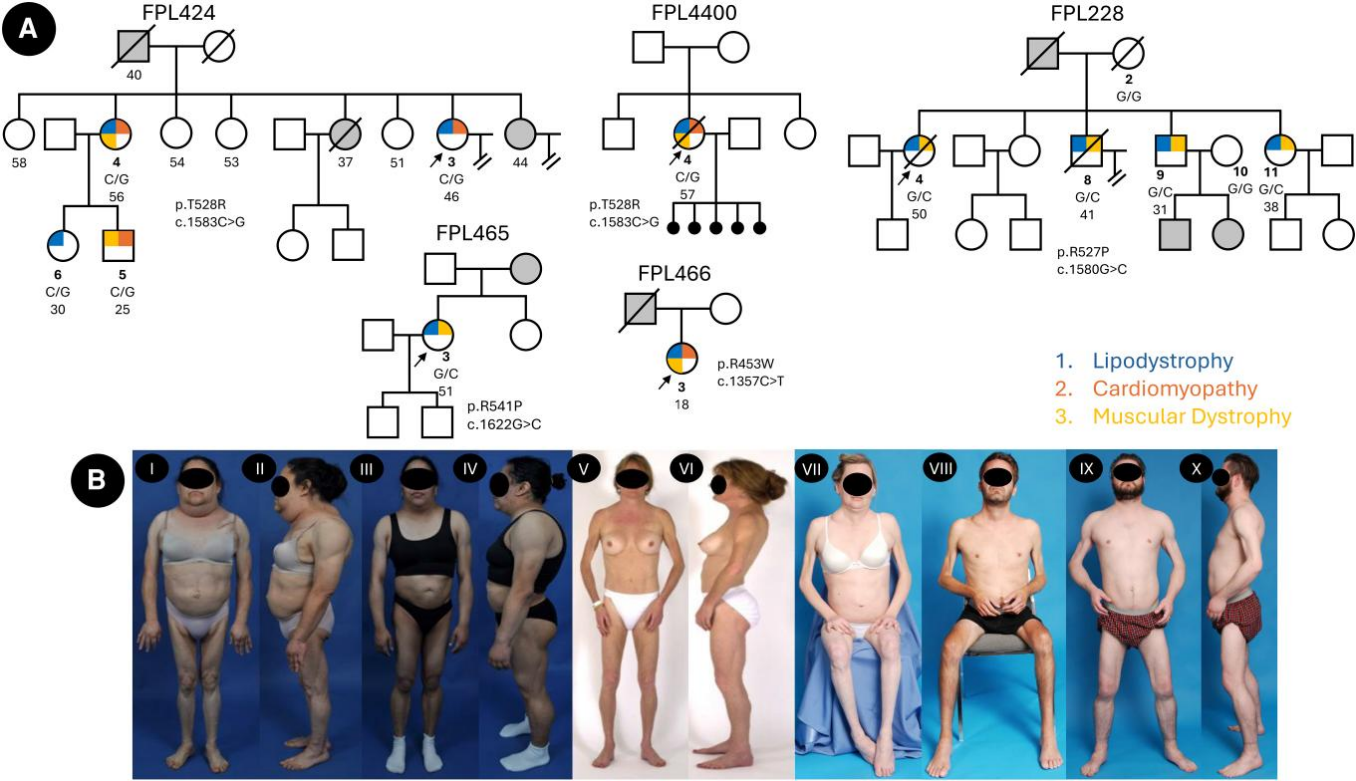
Pedigrees of our patients and clinical features. (A) Pedigrees of our patients with co-occurrence of familial partial lipodystrophy, Dunnigan variety, and Emery-Dreifuss muscular dystrophy. The proband in each family is marked by a slanted arrow. Squares and circles represent males and females, respectively. A diagonal line across the symbol indicates deceased subject. The different color quadrants represent different phenotypes, and the unfilled (white) symbols show those without disease. The gray symbols represent those who reportedly had EDMD and FPLD2 but without confirmation of the genotype. Black dots indicate premature termination of pregnancy. The numbers below the symbols indicate ages in years at last follow-up. (B) Clinical features of our patients with co-occurrence of familial partial lipodystrophy, Dunnigan variety, and Emery-Dreifuss muscular dystrophy. All the probands were females and had increased muscularity and prominent veins in the upper and lower extremities due to marked loss of subcutaneous fat. (I, II) Anterior and lateral views of the patient FPL424.3, a 46-year-old female, demonstrating loss of subcutaneous fat from the arms, legs, and abdomen. Excess fat deposition is evident in the neck (double chin), supraclavicular, dorsocervical, and mons pubis regions. The umbilicus is protruded, and the lateral border of the rectus abdominis is visible due to loss of anterior abdominal fat. She has a scar on the left upper chest from pacemaker placement. (III, IV) Anterior and lateral views of the patient FPL424.6, a 30-year-old female, demonstrating muscular arms and legs. Excess fat deposition is evident in the neck (double chin). Acanthosis nigricans is visible in the neck. (V, VI) Anterior and lateral views of the patient FPL4400.4, a 57-year-old female, demonstrating reduced subcutaneous fat deposition in the abdomen, arms, and legs. Excess fat deposition is evident in the neck (double chin) and supraclavicular region. Lateral borders of rectus abdominis are prominent. She had breast implants. (VII) Anterior view of the patient FPL228.4, a 50-year-old female, demonstrating muscular arms and legs with loss of subcutaneous fat; excess fat deposition is evident in the neck (double chin), supraclavicular region, and protruded umbilicus. She was wheelchair bound and unable to stand. (VIII) Anterior view of the patient FPL228.8, a 40-year-old male, demonstrating thin arms and legs due to loss of subcutaneous fat. Excess fat deposition is evident in the supraclavicular region. He was wheelchair bound and unable to stand. (IX, X) Anterior and lateral views of the patient FPL228.9, a 30-year-old male, with muscular arms and legs, demonstrating loss of subcutaneous fat from the legs and abdomen. Excess fat deposition is evident in the neck. Abbreviations: C/G, heterozygous c.1583C>G *LMNA* variant; C/T, heterozygous c.1357C>T *LMNA* variant; G/C, heterozygous c.1580G>C and c.1622G>C *LMNA* variants.

The description of individual cases follows. Table 1 shows the biochemical and metabolic data of the affected individuals, and their pedigrees and clinical features are shown in Fig. 1. Clinical features of each patient pertaining to AD-EDMD are given in Table 2.

**Table 1:** Clinical features, regional body fat, and metabolic parameters of patients with Emery-Dreifuss muscular dystrophy and Dunnigan-type familial partial lipodystrophy

Patient ID	424.3	424.4	424.5	424.6	4400.4	228.4	228.8	228.9	228.11	465.3	466.3	*P*-value
*LMNA* variant	T528R	T528R	T528R	T528R	T528R	R527P	R527P	R527P	R527P	R541P	R453W	NA
Age, years	46	56	25	30	58	50	40	30	29	51	18	NA
Sex	F	F	M	F	F	F	M	M	F	F	F	NA
Race	White	White	White	White	White	White	White	White	White	White	White	NA
Ethnicity	H	H	H	H	NH	NH	NH	NH	NH	NH	NH	.18
Diabetes mellitus	Y	Y	N	Y	N	N	N	N	N	Y	Y	.06
Dyslipidemia	Y	Y	N	N	N	Y	N	N	Y	Y	Y	.30
Triglycerides, mg/dL	114	164	130	98	250	228	74	195	304	289	285	.19
Cholesterol, mg/dL	162	178	174	146	217	163	209	146	169	200	178	1
LDL-C, mg/dL	100	99	109	89	132	77	66	76	80	112	110	.19
HDL-C, mg/dL	39	46	40	38	42	40	128	31	28	39	19	.53
HbA1c, %	8.5	7.1	6	7.5	6	5.9	5.1	5.6	5.4	7.1	10.6	.10
Glucose, mg/dL	85	101	85	119	88	88	81	85	94	114	228	.03
Leptin, ng/mL	6.0	5.1	6.4	2.8	NA	7.4	NA	8.4	NA	4.6	28.2	.42
Uric acid, mg/dL	6.2	3.7	6.4	6.2	NA	5.1	3.0	5.1	4.8	7.1	7.1	.56
AST, IU/L	20	19	18	41	NA	15	323	38	19	17	122	.49
ALT, IU/L	31	17	32	81	NA	12	76	82	24	18	97	.26
Creatine Kinase, U/L	797	77	798	458	487	139	152	925	531	232	239	.27
Total body fat, %^*a*^	35.3	NA	33	26.8	35.1	38.8	NA	35.6	NA	22.9	38.1	NA
Upper extremities fat, %^*a*^	37.1	NA	36	29.9	41.9	45.7	NA	34.7	NA	22.2	43.7	NA
Lower extremities fat, %^*a*^	28	NA	34.7	17.7	42	42.8	NA	35.8	NA	17.7	38.1	NA
Head fat, %^*a*^	19.9	NA	20.3	18.9	20.3	23.5	NA	24.6	NA	21.0	29.1	NA
Trunk fat, %^*a*^	38.5	NA	32.4	29.8	33.1	38.2	NA	37.1	NA	25.7	37.7	NA
Bone mineral density, g/cm^2*a*^	0.88	NA	1.10	1.02	1.053	1.38	NA	0.93	NA	1.07	1.12	NA
Bone mineral density, Z-score^*a*^	−2.5	NA	−1.0	−1.1	0.1	2.7	NA	−2.9	NA	−0.0	0.4	NA

*P* value indicates comparison between males and females.

Abbreviations: ALT, alanine aminotransferase; AST, aspartate aminotransferase; F, female; H, Hispanic; HbA1c, hemoglobin A1c; HDL-C, high-density lipoprotein-cholesterol; LDL-C, low-density lipoprotein-cholesterol; M, male; N, No; NA, not available; NH, non-Hispanic; Y, yes.

^*a*^Measured by dual-energy X-ray absorptiometry.

**Table 2:** Clinical features of Emery-Dreifuss muscular dystrophy in our patients

Patient ID	424.3	424.4	424.5	424.6	4400.4	228.4	228.8	228.9	228.11	465.3	466.3
*LMNA* variant	T528R	T528R	T528R	T528R	T528R	R527P	R527P	R527P	R527P	R541P	R453W
Age, year	46	56	25	30	57	50	40	30	29	51	18
Sex	F	F	M	F	F	F	M	M	F	F	F
Muscle weakness	Proximal in UL and LL	Proximal and distal in UL and LL	Proximal in UL and LL	No	Proximal and distal In UL and LL	Proximal in UL and LL	Yes	Proximal in UL and LL	Proximal in LL	Proximal in LL	Proximal in UL and LL
Mobility assisted device	No	Yes	No	No	No	Yes	Yes	No	Yes	No	No
Contractures	pes equinus	Elbow, knees	neck	No	Yes	Neck, elbow Achilles	Neck, Achilles	Neck, Elbow, Achilles	Yes	No	No
Arrhythmia	Complete heart block, atrial fibrillation, VT	Atrial standstill, junctional bradycardia	Atrial fibrillation	No	Bradycardia	Atrial fibrillation	No	Atrial fibrillation	No	No	Atrial tachycardia
Cardiomyopathy	Yes	Yes	Yes	No	No	No	No	Yes	No	No	No
ICD/pacemaker	Yes	Yes	No	No	Yes	Yes	No	Yes	No	No	Loop recorder
Scoliosis	Yes	Yes	No	Yes	No	Yes	Bending of cervical spine	No	No	No	No

Abbreviations: F, female; ICD, intracardiac device; LL, lower limb; M, male; UL, upper limb; VT, ventricular tachycardia.

#### FPL424.3

This 46-year-old Hispanic female with hyperlipidemia and diabetes mellitus presented for evaluation of abnormal fat distribution. She had complete heart block, atrial fibrillation, heart failure with reduced left ventricular ejection fraction (LVEF, 44%), and sustained ventricular tachycardia for which cardiac resynchronization therapy defibrillator and Implantable cardioverter-defibrillator were implanted at 40 years of age. As a child, she had a surgical correction for pes equinus. Her medications included empagliflozin. linagliptin, metformin, metoprolol succinate, rivaroxaban, sacubitril-valsartan, spironolactone, and venlafaxine. On examination, acanthosis nigricans was present in the neck, axilla, and groin. She had loss of subcutaneous fat from the arms and legs. There was an increase in subcutaneous fat in the labia majora but no clitoromegaly. She had a round face, double chin, and increased fat in the neck and supraclavicular region with a small dorsocervical fat pad (Fig. 1B, I and II). She was also found to have a low bone density Z-score (−2.5) on the DEXA scan for body fat, but a repeat DEXA for bone density showed osteopenia with a T-score of −1.9 and Z-score of −1.7. Her liver was palpable 5 cm and spleen 3 cm below the right and left costal margins, respectively. She had 3 sisters with lipodystrophy. Her eldest sister (FPL424.4) also has muscular dystrophy with a pacemaker. One of the younger sisters with lipodystrophy died of myocardial infarction at the age of 37 years, and the youngest one has diabetes and a pacemaker (Fig. 1). Her father also had similar phenotype of lipodystrophy and died from heart disease at the age of 40 years.

#### FPL424.4

This 56-year-old sister of patient FPL424.3 had hyperlipidemia diagnosed at age 55 years and diabetes mellitus at 50 years of age. She had atrial standstill and junctional bradycardia for which a pacemaker was inserted at the age of 41 years. Eight years later, a cardiac resynchronization therapy pacemaker-defibrillator was implanted. She had the right occipital lobe infarct at the age of 45 years secondary to atrial fibrillation. She was diagnosed with muscular dystrophy at the age of 48 years associated with cardiomyopathy and heart failure (LVEF 43%). Her medications included metoprolol, baclofen, empagliflozin, lisinopril, spironolactone, atorvastatin, and rivaroxaban. She has been using a mobility assistance device for the last 5 years and needs help with activities of daily living. On examination, there were multiple skin tags in the neck but no acanthosis. She had atrophic breasts and contractures in the left hand and arm. She had loss of subcutaneous fat from the extremities.

#### FPL424.5

This 25-year-old son of patient FPL424.4 was evaluated for lipodystrophy. On examination, his pulse rate was 76 beats per minute and irregular. He had a double chin with mild acanthosis nigricans. There was mild gynecomastia with a small dorsocervical fat pad. His liver was palpable 5 cm below the right costal margin. He complained about difficulty in climbing stairs, and neuromuscular examination showed hip flexion and shoulder abduction strength of 4/5 with absent patellar deep tendon reflexes bilaterally. He was unable to stand from the floor without support, and a neck extension contracture was present. Electrocardiography showed atrial fibrillation with normal ventricular response. Transthoracic echocardiography and cardiac magnetic resonance imaging (MRI) showed a mildly dilated left atrium (LVEF 55%). He underwent cardioversion which was unsuccessful.

#### FPL424.6

This 30-year-old daughter of FPL424.4 was diagnosed with hypertriglyceridemia at 17 years of age. She had scoliosis diagnosed at 7 years of age and left knee surgery for limb length discrepancy at the age of 12 years. She had irregular and heavy menstrual cycles, and her left kidney was congenitally absent. On physical examination, she had acanthosis nigricans in the axilla and groin. There was loss of subcutaneous fat from her arms and legs (Fig. 1B, III and IV), and her liver was palpable 7 cm below the right costal margin. Muscle power was 5/5 in all groups, but deep tendon reflexes were reduced.

#### FPL4400.4

This 58-year-old white female was diagnosed with EDMD at age 35 years. At the age of 6 years, she developed weakness in her lower extremities leading to foot drop and contractures. At the age of 11 years, she developed spinal rigidity and shoulder weakness. Later, intraventricular conduction defects developed with symptomatic bradycardia, and a pacemaker was placed at age 39 years. She also had fat loss from upper and lower extremities in her late teens. She was diagnosed with lipodystrophy at age 42 years. She did not have diabetes mellitus or hypertriglyceridemia. Her medications included warfarin, mesalamine, and conjugated estrogen. She had a history of 3 miscarriages and 2 tubal pregnancies. There was no family history of lipodystrophy or muscular dystrophy. On physical examination, she had a thin trunk and extremities due to loss of subcutaneous fat with excess fat deposition on the face and neck (Fig. 1B, V and VI). She died at age 58 years most likely due to cerebrovascular accident. She had 2 strokes 9 months prior that left her with very limited residual mobility.

#### FPL228.4

This 50-year-old female had difficulty walking and was falling at the age of 3 to 4 years. She had Achilles tendon contractures at 6 years of age and underwent cord lengthening at 7 years. At the time of puberty at 12 years, she noticed prominence of veins in her legs. She also had scoliosis and underwent surgery and had atrial fibrillation. On examination, she had supraclavicular fat pads with a double chin. There was loss of fat from the arms, legs, gluteal region, and abdomen (Fig. 1B, VII). There was no acanthosis or hepatosplenomegaly. She had proximal muscle weakness in both the upper and lower extremities, and tendon reflexes were reduced. There were contractures of the neck, elbow, and thickened Achilles. Scoliosis was present from thoracic to lumbar vertebrae. At the age of 31, she delivered a child through caesarean section. On follow-up, she had a pacemaker-defibrillator. She died at the age of 50 years due to aspiration pneumonia. She and some members of her family were included as EMD4 pedigree in the paper by Bonne et al [7].

#### FPL228.8

This 40-year-old male started developing severe neck contractures at age 18 years, for which he underwent extensive surgery. He also had heel cord lengthening surgery. He had muscular weakness and loss of subcutaneous fat (Fig. 1B, VIII). He had liver problems due to excessive alcohol consumption and died at the age of 40 years.

#### FPL228.9

This 30-year-old male was diagnosed with atrial fibrillation in his 20s and was prescribed antiarrhythmic therapy. Around puberty, he noticed difficulty in standing up from squatting and started developing contractures in the elbows. He had bilateral Achilles tendon lengthening surgery at 26 years of age. On examination, he had contractures in the elbows bilaterally and in the neck, loss of subcutaneous fat from the upper and lower extremities, double chin, and proximal muscle weakness with absent deep tendon reflexes in the upper and lower extremities (Fig. 1B, IX and X). On follow-up, a pacemaker-defibrillator was implanted at the age of 31 years. His Z-score on whole body DEXA scan was −2.9, and we did not find any underlying cause for the low Z-score.

#### FPL228.11

This 29-year-old female noticed fat loss from the arms, legs, hips, and abdomen at the age of 12 years. She had Achilles tendon lengthening surgery at the age of 24 and was diagnosed with hypertriglyceridemia. She also noticed difficulty climbing stairs and tiredness upon running. On examination, she had muscular arms and legs with reduced subcutaneous fat and more fat deposition in the neck. On follow-up, at the age of 38, she was using a power chair and had 2 children.

#### FPL465.3

This 51-year-old female was found to have elevated serum triglycerides at the age of 16 years on routine test and was diagnosed with FPLD2. She had multiple episodes of acute pancreatitis due to extreme hypertriglyceridemia requiring hospitalization. During her second episode of acute pancreatitis, she was diagnosed with diabetes mellitus requiring insulin therapy. She had 2 pregnancies with a history of gestational diabetes, and 1 of the pregnancies was assisted. Current medications included metformin, pancrelipase, pioglitazone, and fenofibrate. She had regular menstrual cycles. She had difficulty climbing the stairs, and her paternal great-grandmother was in a wheelchair by age 40. On examination, she had supraclavicular fat pads, hepatomegaly 5 cm below right costal margin, and muscular extremities with loss of subcutaneous fat. She had proximal muscle weakness as evidenced by inability to sit up from supine position without rolling to side and using arms.

#### FPL466.3

This 18-year-old female was diagnosed with diabetes mellitus 3 months ago, on a routine blood glucose test performed prior to a dental procedure. She had loop recorder implantation for ectopic atrial tachycardia, hypertension, and dyslipidemia. She had menarche at the age of 10 years and regular periods and was currently using estrogen for contraception. Her current medications included metformin and insulin (3 U/kg/day), lisinopril, rosuvastatin, spironolactone, metoprolol, and amitriptyline. Her father had similar body habitus and muscular dystrophy and died at the age of 40 from cardiac complications. On examination, she had loss of fat from the breasts, buttocks, lower limbs, and forearms, preserved fat in the upper arms, back, and abdomen and gain of fat in the face and neck. She had decreased muscle strength (4/5) in both the upper and lower extremities. Ultrasound of the abdomen showed hepatosplenomegaly with diffuse hepatic steatosis and echocardiogram was normal (LVEF 58%).

## Results

**Figure 2:**
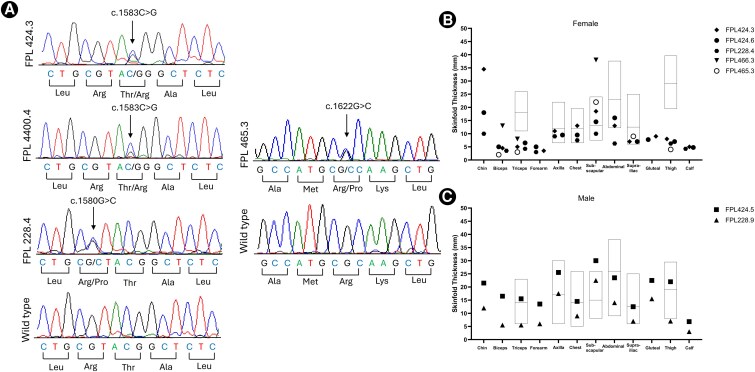
*LMNA* sequence chromatograms and skinfold thicknesses of our patients with familial partial lipodystrophy, Dunnigan variety, and Emery-Dreifuss muscular dystrophy. (A) *LMNA* sequence chromatograms on Sanger sequencing for the probands. FPL424.3 and FPL4400.4 show heterozygous (het) c.1583C>G variant (p.T528R), FPL228.4 shows het c.1580G>C variant (p.R527P), and FPL465.3 shows het c.1622G>C variant (p.R541P). The corresponding wild-type *LMNA* chromatograms are shown below the variants for reference. (B) Skinfold thickness of 5 females and (C) 2 males with familial partial lipodystrophy, Dunnigan type and autosomal dominant Emery-Dreifuss muscular dystrophy measured with standard skinfold calipers. The white bars show 10th to 90th percentile values of normal age-matched males [31] and females [32] with the median value marked by a horizontal line. All females and 1 male (FPL228.9) have the triceps and thigh skinfold thickness values <10th percentile of normal values.

We evaluated 5 families (8 females, 3 males) with FPLD2 and AD-EDMD. All the participants from FPL424 and FPL4400 (4 females, 1 male) had heterozygous missense *LMNA* variant c.1583C>G, (p.T528R) (Fig. 2A); those from the FPL228 family had c.1580G>C (p.R527P) variant, FPL465.3 had c.1622G>C (p.R541P) variant, and FPL466.3 had c.1357C>T (p.R453W) variant. All of these variants were absent from gnomAD, All of US, and UK Biobank, except p.R453W, which was present in 1 out of 829 634 alleles in the All of Us study with a minor allele frequency of 1 × 10^−6^. FPL424.4 did not have any pathogenic variants in *EMD*, *FHL1*, *MYOT*, and *SYNE1* genes on genetic testing from a commercial laboratory. FPL424.5 had 3 variants of uncertain significance in *MYO18B* gene, and FPL424.6 tested negative for *EMD* and *FHL1* genes. The pathogenic variant in family FPL228 was reported earlier by Bonne et al [7] as family EMD4.

We compared the prevalence of metabolic complications and metabolic variables in 3 affected males and 8 affected females. Five females were diagnosed with diabetes mellitus, and 2 had prediabetes, while none of the males had diabetes; however, this difference was not statistically significant (*P* = .06). Similarly, the prevalence of dyslipidemia was higher in females as compared with males but did not achieve statistical significance (*P* = .30). The females had significantly higher fasting glucose compared to the males (*P* = .03). None of the other metabolic variables were significantly different between the males and females. Elevated serum creatine kinase levels were present in all patients except FPL424.4, FPL228.4, and FPL228.8. Increased serum liver enzyme levels were noted in FPL424.6 and FPL466.3, most likely due to steatohepatitis (Table 1).

A total of 7 out of 11 participants had some degree of arrhythmia, and 5 participants had a pacemaker-defibrillator implanted. Five females and 3 males had contractures in the neck, elbow, and ankle, and 3 females (FPL424.4, FPL228.4, and FPL228.11) used mobility assistance devices (Table 2).

All females and FPL228.9 had the triceps and anterior thigh skinfold measurements <10th percentile of normal values, while the skinfold thickness in the thigh and triceps in FPL424.5 were above the median values. However, all participants had truncal skinfold measurements between 10th to >90th percentile (Fig. 2B and 2C).

**Figure 3:**
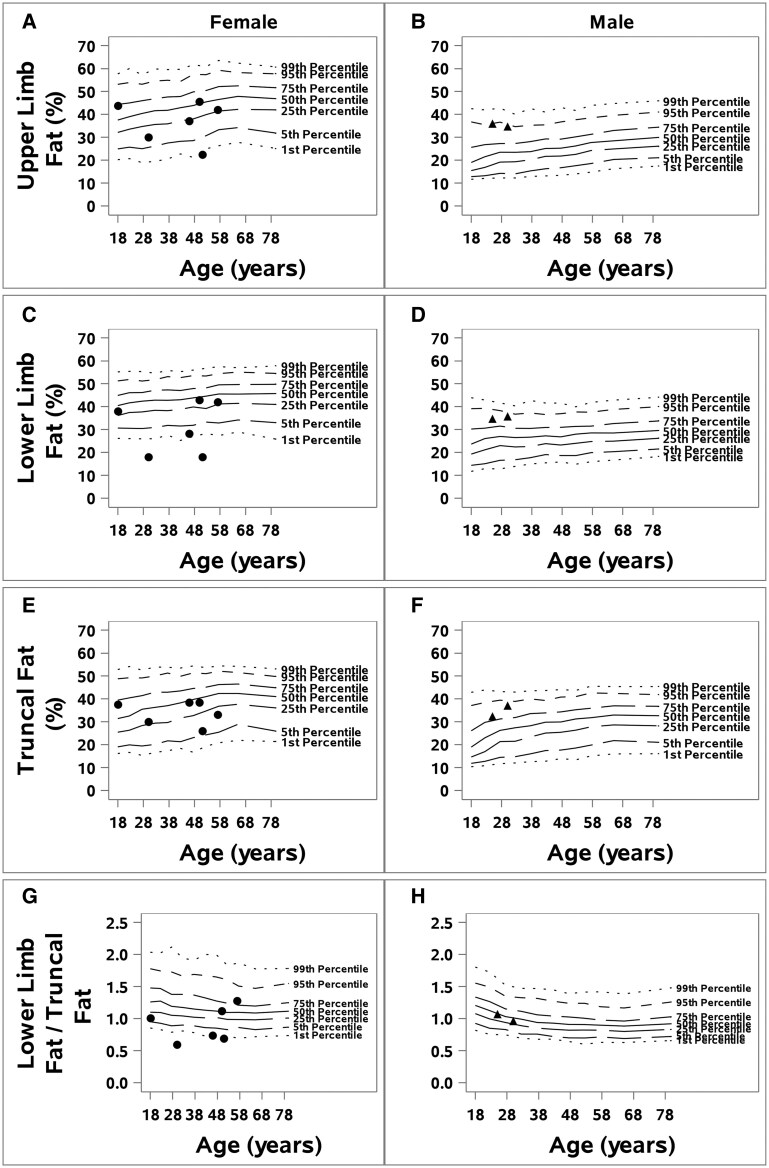
Comparison of regional body fat by dual-energy X-ray absorptiometry scan of females and males with familial partial lipodystrophy, Dunnigan type and autosomal dominant Emery-Dreifuss muscular dystrophy with the National Health and Nutrition Examination Survey (NHANES) controls. (A) The upper limb fat (%) was between the 5th and 75th percentiles in most of the affected females except 1 (FPL 465.3), who had it below the 1st percentile. (B) Both males had the upper limb fat (%) at almost the 95th percentile. (C) Two females had lower limb fat (%) ≤ 1st percentile of NHANES, while the other four had it between the 1st and 50th percentiles. (D) Both males had lower limb fat (%) above the 75th percentile. (E) Truncal fat ranged between the 5th and 75th percentiles in affected females. (F) Truncal fat was between the 75th and 95th percentiles of NHANES in the 2 affected males. (G) The ratio of lower limb fat/truncal fat was ≤1st percentile of NHANES in 3 affected females and between 5th to 75th percentiles in the other 3. (H) Both males had a ratio of lower limb fat/truncal fat between the 25th and 50th percentiles.

Lower limb fat percentage on DXA scan was below the 1st percentile of the NHANES controls in 2 females (FPL424.6 and FPL465.3), whereas it was between the 1st and 50th percentile in all other females. However, 2 males (FPL424.5 and FPL228.9) had lower limb fat percentage on DXA scan above the 75th percentile of the NHANES (Fig. 3).

## Discussion

Specific heterozygous pathogenic variants in *LMNA* present pleiotropic effects and cause a spectrum of distinct disorders such as AD-EDMD, FPLD2, limb-girdle muscular dystrophy, atypical progeroid syndrome, and Hutchinson-Gilford progeria syndrome, among others [7-10, 12, 14]. We and others have previously reported families with multisystem dystrophy with overlapping clinical features of FPLD2, limb-girdle muscular dystrophy, and progeroid manifestations due to missense heterozygous *LMNA* variants [23, 24]. However, in those reports, the patients had only mild myopathy. There is a paucity of reports of overlapping syndromes of AD-EDMD and FPLD2 in the literature. More than 150 cases have been reported in the literature as AD-EDMD due to heterozygous pathogenic variants in *LMNA*, with the most common variants being c.1357C>T; (p.R453W) and c.16C>T; (p.Q6*) [33-36]. Also, more than 300 patients have been reported with FPLD2 [11], with the most common *LMNA* variants being c.1444C>T (p.R482W) and c.1445G>A (p.R482Q). Despite so many reports of individuals with AD-EDMD and FPLD2, only 2 pedigrees were reported previously with overlapping features of AD-EDMD and FPLD2 [25]. Our report of 5 unique families with the heterozygous missense variants in *LMNA*, which caused variable presentation of FPLD2 and AD-EDMD among the family members, now suggests that such overlap is not rare but may be often missed due to lack of recognition of FPLD2 among patients presenting with AD-EDMD.

A total of 8 females and 3 males from 5 families were affected. Interestingly, on clinical examination and skinfold thickness measurements, the phenotype of FPLD2 in the affected females was as severe as seen in those with “typical” FPLD2. In addition, 1 male patient (FPL228.9) showed a typical FPLD2 phenotype; however, the other young male (FPL424.5), who harbored the p.T528R variant, did not show significant peripheral fat loss. Nevertheless, he had prediabetes, low serum levels of HDL-C, acanthosis nigricans, dorsocervical fat pad, and hepatomegaly, all suggestive of underlying metabolic dysfunction.

Consistent with our previous findings of higher prevalence of metabolic complications among females with FPLD2 compared to males [37], a similar trend was observed in patients with both FPLD2 and AD-EDMD in this cohort, although the differences in the prevalence of diabetes and dyslipidemia did not reach statistical significance [37]. The females with FPLD2 and AD-EDMD also had significantly higher fasting plasma glucose compared to the males (*P* = .03).

Another interesting finding was normal amount of lower extremity fat on DXA scan in all female patients, except in FPL424.6 and FPL465.3 (18%), both of whom clinically did not have muscular dystrophy. We have previously shown that adult females with typical FPLD2 have lower extremity fat <1st percentile of the age- and sex-matched values from NHANES [26]. However, none of those FPLD2 patients had features of muscular dystrophy [26]. The corresponding 1st percentile values of lower extremity fat for the age groups for FPL424.3, FPL4400.4, FPL228.4, and FPL466.3 are 27.4%, 27.5%, 28.0%, and 26.2%, respectively [26], and none of them had lower extremity fat below the 1st percentile of normal. However, all our female patients with typical FPLD2 phenotype had anterior thigh skinfold thickness values much below the 10th percentile of normal values [32]. We believe that this discrepancy between skinfold thickness measurement and DXA-based determination of extremity fat is likely due to associated EDMD causing steatosis in lower extremity muscles. This is supported by the previous report of van der Kooi et al [25], who reported marked steatosis of muscles in the lower extremity on computerized tomography imaging of a female patient with AD-EDMD and FPLD2. Therefore, the anthropometric measures (eg, skinfold thickness) may be more reliable in patients with overlapping features of FPLD2 and AD-EDMD. Future studies in such patients should incorporate MRI or computed tomography imaging to directly assess muscle fat infiltration as well as subcutaneous fat loss.

Interestingly, 7 patients, 2 males and 5 females, with the heterozygous p.T528R variant in *LMNA* have been previously reported to have AD-EDMD [38-42]. Just like some of our patients, a young 29-year-old male [41] was wheelchair bound by the age of 17 years and had a pacemaker implantation, and another 19-year-old female underwent surgery for pes equinus and rehabilitation to improve mobility [42]. However, these investigators failed to report lipodystrophy among these patients. It is likely that the diagnosis of FPLD2 was missed by the investigators since their primary specialty was neurology or cardiology and they may have focused on the primary diagnosis. Also, the 2 male patients, just like the affected male in our pedigree, either may not have had clinical lipodystrophy or the diagnosis may have been missed due to “ascertainment bias” [37].

Interestingly, some individuals from the FPL228 family have been reported previously in association with AD-EDMD as EMD4 [7]. Three years later, 3 affected individuals from another family (EMD6) in the paper [7] and 1 more case reported with similar variants (p.R527P) were also found to have lipodystrophy along with cardiac and muscular abnormalities [25]. However, the report of Van der Kooi et al [25] specifically stated a lack of lipodystrophy in the EMD4 pedigree. We have subsequently conducted an in-depth investigation for lipodystrophy phenotype among the affected patients from the same family and have found clear evidence of FPLD2 and associated metabolic abnormalities. This further supports our contention that previous investigators may have failed to recognize the diagnosis of FPLD2 in patients with AD-EDMD.

Another interesting observation was the heterogeneous presentation of FPLD2, AD-EDMD, and cardiomyopathy among patients within the same family, which could be due to variable expression of mutant lamins A and C among different tissues, sexual dimorphism, age difference, or other modifier genetic variants, which remain to be identified. Two patients (FPL424.4 and FPL228.4) had almost normal serum creatine kinase levels, which could be due to their reduced muscle mass and because they were wheelchair bound.

Finally, our report brings attention to the frequent co-occurrence of FPLD2 and AD-EDMD. In the future, patients with AD-EDMD due to *LMNA* variants should be evaluated for lipodystrophy and metabolic complications. It is possible that more AD-EDMD patients will be identified to have the FPLD2 phenotype by careful screening using anthropometry including skinfold thickness measurements and whole-body MRI for assessing regional body fat distribution, as DXA scan may not accurately estimate subcutaneous fat in the lower extremities of AD-EDMD patients.

## Data Availability

Some or all datasets generated during and/or analyzed during the current study are not publicly available but are available from the corresponding author on reasonable request.
